# E2F1-induced upregulation of long noncoding RNA LINC00668 predicts a poor prognosis of gastric cancer and promotes cell proliferation through epigenetically silencing of CKIs

**DOI:** 10.18632/oncotarget.6745

**Published:** 2015-12-23

**Authors:** Erbao Zhang, Dandan Yin, Liang Han, Xuezhi He, Xinxin Si, Wenming Chen, Rui Xia, Tongpeng Xu, Dongying Gu, Wei De, Renhua Guo, Zhi Xu, Jinfei Chen

**Affiliations:** ^1^ Department of Biochemistry and Molecular Biology, Nanjing Medical University, Nanjing, Jiangsu, PR China; ^2^ Central Laboratory, the Second Affiliated Hospital of Southeast University, Nanjing, Jiangsu, PR China; ^3^ Department of Oncology, Xuzhou Central Hospital, Affiliated Xuzhou Hospital, College of Medicine, Southeast University, Xuzhou, Jiangsu, PR China; ^4^ Department of Oncology, First Affiliated Hospital of Nanjing Medical University, Nanjing, Jiangsu, PR China; ^5^ Department of Oncology, Nanjing First Hospital, Nanjing Medical University, Nanjing, Jiangsu, PR China; ^6^ Collaborative Innovation Center for Cancer Personalized Medicine, Nanjing Medical University, Nanjing, Jiangsu, PR China; ^7^ Departments of Pathology, First Affiliated Hospital of Nanjing Medical University, Nanjing, Jiangsu, PR China

**Keywords:** E2F1, LINC00668, cell cycle, proliferation, gastric cancer

## Abstract

Recently, long noncoding RNAs (lncRNAs) have been shown to have important regulatory roles in human cancer biology. By utilizing publicly available lncRNAs expression profiling data and integrating analyses, we screened out LINC00668, whose expression is significantly increased and correlated with outcomes in gastric cancer (GC). Further experiments revealed that LINC00668 knockdown significantly repressed proliferation, both in *vitro* and in *vivo*. Mechanistic investigations showed that LINC00668 was a direct transcriptional target of E2F transcription factor 1 (E2F1). We further demonstrated that LINC00668 was associated with PRC2 and that this association was required for epigenetic repression of cyclin-dependent protein kinase inhibitors (CKIs), including p15, p16, p21, p27 and p57, thus contributing to the regulation of the gastric cancer cell cycle. Our results suggest that E2F1-activated LINC00668, as a cell cycle regulator, enriches the mechanistic link between lncRNA and the E2F1-mediated cell cycle regulation pathway and may serve as a candidate prognostic biomarker and target for new therapies in human gastric cancer.

## INTRODUCTION

Gastric cancer (GC) is the most common gastrointestinal malignancy in East Asia, Eastern Europe, and parts of Central and South America [1]. Gastric carcinogenesis is a complicated biological process that results from the dysregulation of many tumor-related genes. Therefore, identification of new biomarkers for GC and a better understanding of molecular mechanisms underlying gastric carcinogenesis will improve diagnosis and treatment of GC.

With the development of whole-genome sequencing technology, it was determined that protein-coding sequences occupy less than 2% of the human genome [2]. Long noncoding RNAs (lncRNAs) are a class of transcripts longer than 200 nucleotides with limited protein coding potential [3]. Recently, many studies have shown that lncRNAs could play critical roles in many biological processes, including cellular development, differentiation, and so on [4–9]. In addition, lncRNAs may mediate oncogenic or tumor-suppressing effects and may be cancer biomarkers and therapeutic targets [10–13]. For instance, HOTAIR could be involved in transcriptional repression of HOX loci and promote breast metastasis by binding to the PRC2 (Polycomb Repressive Complex 2) [12]. In addition to regulation of transcription levels, lncRNAs could also serve as a ‘sponge’ to titrate microRNAs, thus participating in post-transcriptional processing [8, 14]. Our previous study showed that HOTAIR could also function as a competing endogenous RNA by sponging miR-331-3p in gastric cancer [15].

Though a small portion of lncRNAs have been functionally characterized, many members in the class remain uncharacterized [16]. In our present study, we analyzed publicly available lncRNAs expression profiling data of gastric cancer and identified LINC00668. LINC00668 has only one transcript, a 1751bp long noncoding RNA, according to the NCBI (Gene, NR_034100.1). LINC00668 was up-regulated in GC tissues and served as an independent predictor for overall survival. In addition, LINC00668 was a direct transcriptional target of E2F1, and LINC00668 could regulate gastric cancer cell proliferation both *in vitro* and in vivo. Moreover, we also found that LINC00668 plays a key role in the gastric cancer cell cycle by epigenetically silencing CDK inhibitors by binding to PRC2, which could in part account for LINC00668-mediated cell growth regulation. LINC00668may serve as a candidate target for new therapies in human gastric cancer.

## RESULTS

### Expression of LINC00668 is upregulated in human gastric cancer tissues and correlates with poor prognosis

**Figure 1:**
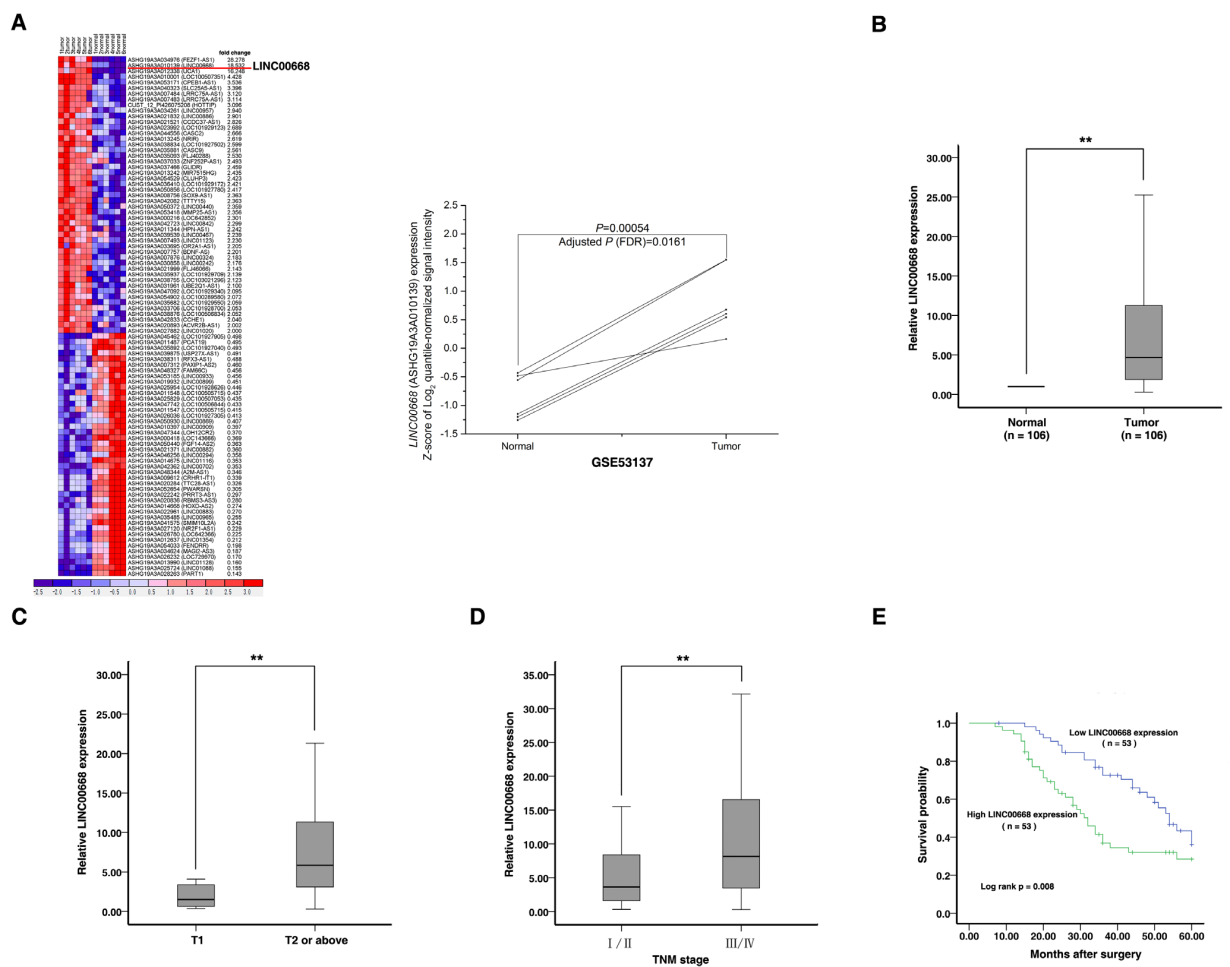
Screening LINC00668 by bioinformatics analysis and its expression in GC tissues and clinical parameters **A.** Raw microarray data were downloaded from GEO Datasets (GSE53137) that described the lncRNA profiles in 6 pairs of human gastric cancer and the corresponding adjacent nontumorous tissues. Then, normalized signal data were downloaded and z-score-transformed. **B.** Relative expression of LINC00668 in GC tissues (*N* = 106) compared with the corresponding non-tumor tissues (*N* = 106). LINC00668 expression was examined by quantitative real-time PCR (qRT-PCR) and normalized to *β-actin* expression. The results are presented as the fold-change in tumor tissues relative to normal tissues. **C.** and **D.** A greater amount of LINC00668 was positively correlated with advanced invasion depth and the TNM stage. **E.** Patients with high levels of LINC00668 expression showed reduced survival times compared with patients with low levels of LINC00668 expression.**, *P* < 0.01.

To obtain differentially expressed lncRNA in gastric cancer, raw microarray data were downloaded from GEO Datasets (GSE53137), which described the lncRNAs profiles in 6 pairs of human gastric cancer and the corresponding adjacent nontumorous tissues [17]. Normalized signal data were then downloaded and z-score-transformed. As show in Figure 1A, we found that LINC00668 was the highest upregulated lncRNA in gastric cancer. To validate the expression results from microarray, we detected the level of LINC00668 in 106 paired GC tissues and adjacent normal tissues by qRT-PCR. As shown in Figure 1B, LINC00668 expression was significantly up-regulated in 85.8% (91 of 106) GC tissues (13.1509±26.68957, *p* < 0.01). Next, we used *T*-tests to examine the correlation between LINC00668 expression and clinic-pathological factors in patients. There were obvious positive correlations between increased LINC00668 levels and advanced invasion depth (14.7835±28.43861 *vs* 3.2467±4.57529, *p* < 0.001) and TNM stage (21.9057±37.35990 *vs* 6.4390±9.87805, *p* = 0.009) (Figure 1C and 1D). Furthermore, we divided the samples into high (above the mean, *n* = 53) and low (below the mean, *n* = 53) LINC00668 expression groups according to the median value of LINC00668 levels. Chi-square tests were performed to evaluate clinic-pathological factors between the two groups. As shown in Table 1, LINC00668 levels were also correlated with tumor invasion depth (*p* = 0.002) and TNM stage (*p* = 0.006). No relationships between LINC00668 expression and other factors, e. g. sex, age or histological grade were found in our study.

**Table 1:** The clinic-pathological factors of GC patients

Clinical factors	Expression of LINC00668		*p* value*
	low	high	
Sex			0.696
male	30	28	
female	23	25	
Age			0.846
≤60	25	26	
>60	28	27	
Histological grade			0.331
Low or undiffer	25	30	
middle or high	28	23	
Invasion depth(T)			0.002**
T1	13	2	
T2 or above	40	51	
Lymph node metastasis (N)			0.326
N0	25	20	
N1 or above	28	33	
TNM stage			0.006**
I/II	37	23	
III/IV	16	30	

* chi-square test

** *p* < 0.01

To determine the relationship between LINC00668 expression and prognosis, Kaplan-Meier analysis was used to evaluate the effects of LINC00668 expression on overall survival (OS). As a result, overexpression of LINC00668 predicted a poor prognosis in patients with GC (*p* = 0.008) (Figure 1E). Univariate analysis identified three prognostic factors: lymph node metastasis (N0, N1 or above), TNM stage (I/II, III/IV) and LINC00668 expression. Multivariate analysis further revealed that LINC00668 expression could be regarded as an independent predictor for overall survival in patients with GC (*p* < 0.001), as well as TNM stage (*p* = 0.003) and lymph node metastasis (*p* = 0.001) (Table 2).

**Table 2:** Univariate and multivariate analysis of clinic pathologic factors for overall survival in 106 patients with GC

Risk factors	Univariate analysis			Multivariate analysis		
	HR*	*p* value	95% CI	HR	*p* value	95% CI
LINC00668 expression	1.024	<0.001**	1.015~1.033	1.018	<0.001**	1.009~1.027
Lymph node metastasis (N0, N1 or above)	2.983	<0.001**	1.680~5.294	2.286	0.001**	1.334~3.917
TNM stage (I/II, III/IV)	2.824	<0.001**	1.689~4.721	2.643	0.003*	1.462~4.776
Histological grade (low, middle or high)	0.756	0.279	0.456~1.254			
Age (≤60, >60)	1.151	0.584	0.695~1.907			
Invasion depth (T1, T2 or above)	1.95	0.122	0.837~4.544			
Sex (male, female)	1.373	0.227	0.821~2.296			

* HR hazard ratio

* *p* < 0.05

** *p* < 0.01

### LINC00668 is activated by E2F1

**Figure 2:**
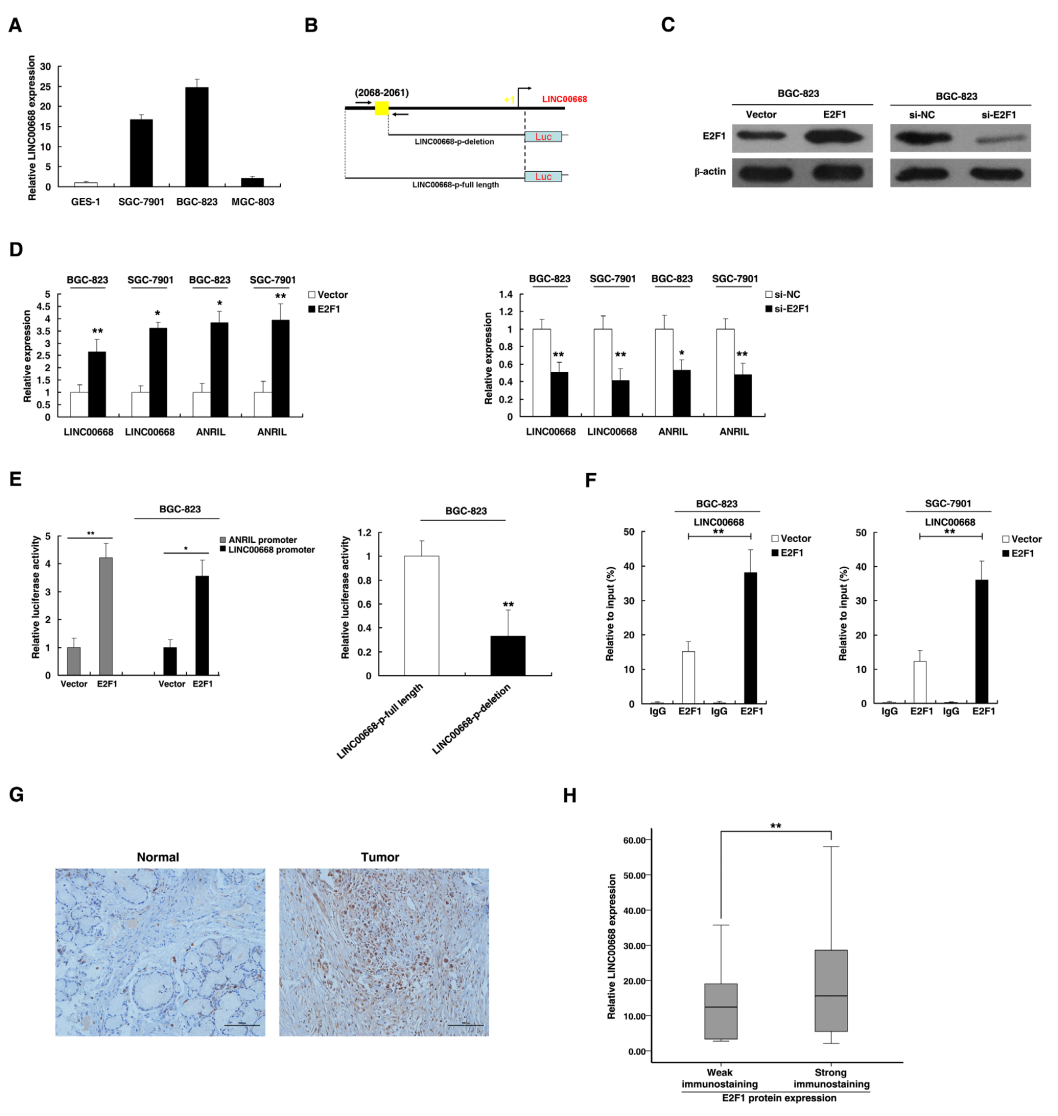
LINC00668 is a direct target of E2F1 **A.** Analysis of LINC00668 expression levels in GC cell lines (SGC-7901, BGC-823 and MGC-803) compared with the normal gastric epithelium cell line (GES-1) by qRT-PCR. **B.** Description of E2F1 binding sites in the promoter region of LINC00668. The position of ChIP primers **F.** was indicated by arrows. **C.** Western blot assays detected the expression of E2F1 after the transfection of the overexpression plasmid and si-RNA. **D.** The expression of LINC00668 and ANRIL was determined in E2F1 and si-E2F1 systems in BGC-823 and SGC-7901 cells. **E.** The luciferase assays were performed to detect the promoter activity of LINC00668. Induction of LINC00668 promoter activity by E2F1 in BGC-823 cells. Then, deletion analysis of the promoter activity was performed to determine the role of the E2F1 site in E2F1-mediated regulation of LINC00668. **F.** Enrichment of E2F1 in the LINC00668 promoter after E2F1 overexpression. E2F1 was immunoprecipitated, and the promoter region containing E2F1-binding sequences were quantified by qRT-PCR. The ChIP primers are detailed in Supplementary Table S3. **G.** Immunohistochemistry was used to detect the expression of the E2F1 protein in GC and corresponding non-tumor tissues. Bar, 100μm. **H.** The immunoreactivity of the E2F1 protein in GC tissues showed a statistically significant positive correlation with the relative level of LINC00668 expression. Error bars indicate the means±S.E.M. *P < 0.05, **P < 0.01. n.s., not significant.

To explore the mechanism of high expression of LINC00668, we examined the LINC00668 expression levels in gastric cancer cell lines. As shown in Figure 2A, gastric cancer cell lines expressed higher levels of LINC00668 compared to normal gastric epithelium cell line (GES-1). Then, based on several computer algorithms (ChIPbase: http://deepbase.sysu.edu.cn/chipbase/, Jaspar: http://jaspar.genereg.net/, and TFSEARCH: http://www.cbrc.jp/research/db/TFSEARCH.html), we performed a computational screen and detected the presence of E2F1 binding sites in the promoter region of LINC00668 (Figure 2B). We addressed whether overexpression of LINC00668 is mediated by E2F1. The expression of E2F1 was up-regulated by an over-expression plasmid of E2F1 and downregulated by siRNA targeting E2F1. To avoid off-target effects and ensure the efficiency of interference, we used an effective interference target sequence of E2F1 from a previous study [18] (Figure 2C). LINC00668 levels were significantly induced in BGC-823 and SGC-7901 cells transfected with an E2F1 over-expression plasmid. In addition, the bona fide E2F1 target lncRNA ANRIL was also induced [19, 20] (Figure 2D). We next sought to investigate whether LINC00668 expression is induced by endogenous E2F1. To this end, siRNA-mediated knockdown of E2F1 resulted in marked decreases in LINC00668 and ANRIL expression levels (Figure 2D).

To determine whether E2F1 directly transcriptionally regulates LINC00668, we cloned the promoter region of LINC00668 into a luciferase reporter plasmid. As shown in Figure 2E, luciferase assays showed that E2F1 induced the promoter activity of LINC00668, which was comparable with the induction of the ANRIL promoter. To further determine the function of the E2F1 binding sites, we made deletions at the promoter of LINC00668 (Figure 2B and 2E). After transfection of E2F1 expression plasmid, the deletion (not containing the E2F1 binding sites) caused significant reduction of promoter activity compared to the full-length promoter construct (Figure 2E). To determine whether E2F1 can directly bind to sites of the LINC00668 promoter *in vitro*, chromatin immunoprecipitation (ChIP) experiments was used to validate that E2F1 could indeed bind to the promoter region of LINC00668. Overexpression of E2F1 resulted in the occupancy of LINC00668 locus (Figure 2F). To further confirm the relationship between E2F1 and LINC00668, immunohistochemistry was used to detect the expression levels of E2F1 protein in 30 paired GC and corresponding non-tumor tissues samples. All of the tumors showed positive immunostaining of E2F1 protein: 7 of 30 GC cases (23%) showed weakly positive staining and 23 GC cases (77%) showed strongly positive staining. In contrast, all of the corresponding non-tumor tissues showed negative or weakly positive immunostaining of E2F1 protein. The representative results were shown in Figure 2G. E2F1 was upregulated in GC tissues. Further analysis showed that the expression of LINC00668 is positively correlated with E2F1 protein levels in GC tissues (Figure 2H).

### LINC00668 promotes gastric cancer cell proliferation by accelerating cell cycle

E2F1 plays pivotal roles in cell cycle progression [21]. In addition, overexpression of E2F1 was observed in various types of tumors, including GC [22, 23]. The above study showed that LINC00668 was a direct target of E2F1. Therefore, we hypothesized that LINC00668 may play a distinct role in the cell cycle of gastric cancer.

**Figure 3:**
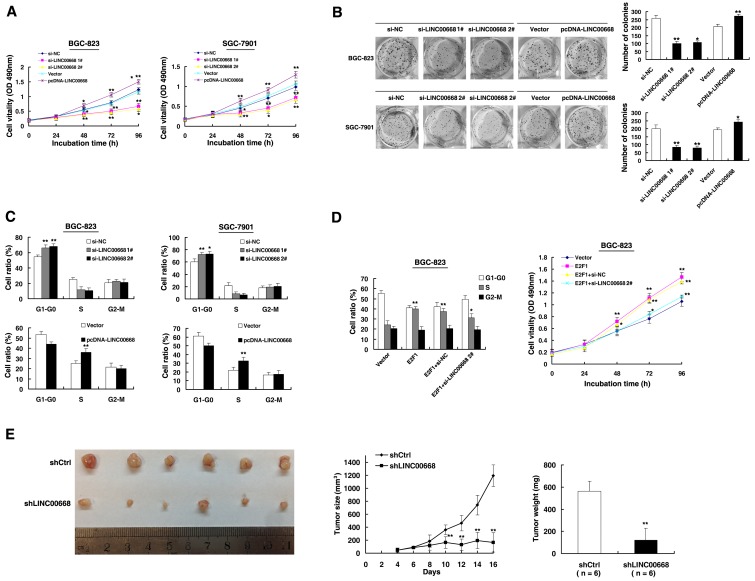
LINC00668 regulates gastric cancer cell proliferation both *in vitro* and *in vivo* **A.** MTT assays were performed to determine the cell proliferation of BGC-823 and SGC-7901 cells after the transfection of the overexpression plasmid and si-RNA of LINC00668. **B.** Representative results of the colony formation of BGC-823 and SGC-7901 cells transfected with the overexpression plasmid and si-RNA of LINC00668. **C.** At 48 h after transfection, the cell cycle was analyzed by flow cytometry. The bar chart represents the percentage of cells in G1-G0, S, or G2-M phase, as indicated. **D.** BGC-823 transfected with Vector/E2F1/E2F1_+_ si-NC and transfected with E2F1 followed by transfection with si-LINC00668. Forty-eight hours after transfection, the cells were analyzed by flow cytometry and MTT assays. **E.** Scramble or shLINC00668 was transfected into BGC-823 cells, which were injected in nude mice (*n* = 6), respectively. Tumor volumes were calculated after injection every two days. Bars indicate the SD. Tumor weights are represented as the means of tumor weights ±SD. *P < 0.05, **P < 0.01.

To test this, we used two effective interference target sequences of LINC00668. qPCR assays revealed that LINC00668 expression was significantly decreased (Figure S1A). LINC00668 expression was upregulated by an expression plasmid of LINC00668 (Figure S1B). MTT assays showed that knockdown of LINC00668 expression significantly inhibited cell proliferation compared to control cells. In contrast, overexpressed LINC00668 could promote cell proliferation (Figure 3A). Similarly, results of colony-formation assays revealed that clonogenic survival was significantly decreased following inhibition of LINC00668. In addition, overexpression of LINC00668 could boost the number of clones (Figure 3B). Next, flow cytometric analysis was performed to further examine whether LINC00668 affected the proliferation of GC cells by altering cell-cycle progression. The results revealed that cell-cycle progression of BGC-823/si-LINC00668 cells was significantly stalled at the G1-G0 phase compared to cells transfected with si-NC. Overexpressed LINC00668 could promote S phase process. Similar results were observed in the SGC-7901 cell line (Figure 3C). Moreover, to further confirm the regulation between E2F1 and LINC00668, we performed rescue experiments. Co-transfection (E2F1 expression plasmid and si-LINC00668) could partially abrogate E2F1 induced cell cycle acceleration and proliferation promotion (Figure 3D). To confirm whether LINC00668 affected tumorigenesis, shCtrl/shLINC00668 transfected BGC-823 cells were inoculated into nude mice. As shown in Figure 3E, tumor growth in the shLINC00668 group was significantly slower than that in the scrambled vector group. Up to 16 days after injection, the average tumor weight in the shLINC00668 group was lower than in the control group.

### LINC00668 facilitates gastric cancer cell proliferation and cell cycle by binding to PRC2

**Figure 4:**
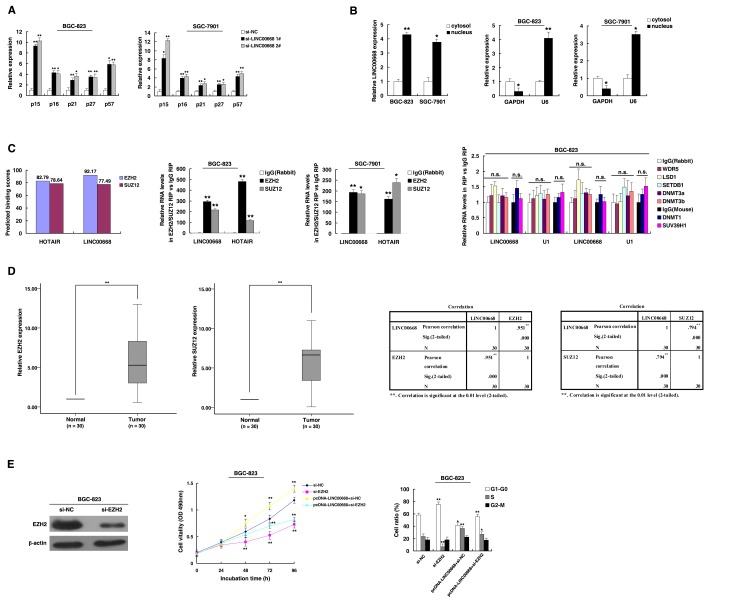
LINC00668 regulates gastric cancer cell proliferation and the cell cycle by specifically binding to PRC2 **A.** Expression of p15, p16, p21, p27 and p57 were determined after knockdown LINC00668 by qRT-PCR assays. **B.** LINC00668 nuclear localization, as identified using qRT-PCR in fractionated BGC-823 and SGC-7901 cells. After nuclear and cytosolic separation, the RNA expression levels were measured by qRT-PCR. GAPDH was used as a cytosolic marker, and U6 was used as a nuclear marker. **C.** Bioinformatics were used to predict the possible interaction of LINC00668 and PRC2. RIPs experiments were performed, and the coprecipitated RNA was subjected to qRT-PCR for LINC00668. The fold enrichment of LINC00668 in RIPs is relative to its matching IgG control RIP. **D.** According to qRT-PCR assays, the level of EZH2 and SUZ12 was upregulated in 30 pairs of GC tissues. The level of EZH2 and SUZ12 in GC tissues showed a statistically positive correlation with the relative level of LINC00668 expression (*N* = 30). **E.** BGC-823 transfected with si-NC/si-EZH2/pcDNA-LINC00668 + si-NC and transfected with LINC00668 followed by transfection with si-EZH2. Forty-eight hours after transfection, the cells were analyzed by MTT assays and flow cytometry. *P < 0.05, **P < 0.01.

To explore the fact that LINC00668 plays a role in G0/G1 arrest, we investigated the expression of CKIs, and the results showed that p15, p16, p21, p27 and p57 were all increased with knockdown of LINC00668 (Figure 4A). To further study the mechanism of its regulation of the gastric cancer cell cycle, we measured LINC00668 expression in nuclear and cytosolic fractions by qRT-PCR. We found a considerable increase in LINC00668 expression in the nucleus *versus* the cytosol (Figure 4B), thus suggesting that LINC00668 may play a major regulatory function at the transcriptional level.

Recent studies have reported that a significant number of lncRNAs have been shown to function in cooperation with chromatin modifying enzymes to promote epigenetic activation or silencing of gene expression [24]. lncRNAs recruit polycomb-group proteins to target genes. Approximately 20% of all human lncRNAs have been shown to physically associate with PRC2, suggesting that lncRNAs may have a general role in recruiting polycomb-group proteins to their target genes [25]. PRC2, a methyltransferase that is composed of EZH2, SUZ12 and EED, can catalyze the di- and trimethylation of lysine residue 27 of histone 3 (H3K27me3), thus epigenetically suppressing gene expression [26]. In addition, aberrations in PRC2 are closely related to carcinogenesis [27]. Thus, we hypothesized that LINC00668 might affect gene expression in such a manner. To test this, first, according to a description by Lu Q et al. [28], we used bioinformatics to predict this possibility (http://bioinfo.bjmu.edu.cn/lncpro/). As shown in Figure 4C, using lncRNA HOTAIR as a positive control, the predicted binding scores between LINC00668 and PRC2 (EZH2/SUZ12) were fairly high. To validate this possibility, RNA immunoprecipitation (RIP) assays showed that the endogenous LINC00668 was enriched in the anti-EZH2 RIP fraction relative to the input compared to the IgG fraction. Moreover, using an antibody specific to SUZ12, another member of the PRC2 complex, we also observed that LINC00668 was enriched in the anti-SUZ12 RNA-IP fraction (Figure 4C). The endogenous lncRNA HOTAIR, which binds PRC2, was used as positive control [12]. In addition, we employed RIP with antibodies against a panel of chromatin modifiers related to tumorigenesis, including WDR5, LSD1, SETDB1, SUV39H1, DNMT1, DNMT3a, and DNMT3b [24, 29, 30]. As shown in Figure 4C, there was no substantial enrichment in RIPs of these chromatin modifiers relative to respective IgG control, U1 was used as negative control. Our results demonstrated that LINC00668 could specifically bind to PRC2, but not with other chromatin modifiers.

Furthermore, to investigate the roles of EZH2/SUZ12 in gastric cancer, we performed qRT-PCR analysis and found that EZH2/SUZ12 expression was significantly increased in 30 pairs of gastric cancer tissues. Further analysis demonstrated that LINC00668 was positively correlated with EZH2/SUZ12 expression (Figure 4D). Furthermore, knockdown of EZH2 (the key catalytic subunit of PRC2 histone methyltransferase) could induce growth inhibition and G1-G0 phase arrest (Figure 4E). Co-transfection (LINC00668 expression plasmid and si-EZH2) could partially abrogate LINC00668 caused proliferation promotion and cell cycle acceleration. These results indicated that LINC00668 may inhibit expression of CKIs in a PRC2-dependent manner.

### LINC00668 was required for the epigenetic repression of cyclin-dependent protein kinase inhibitors (CKIs) by binding to PRC2, thus accelerating cell cycle in gastric cancer

**Figure 5:**
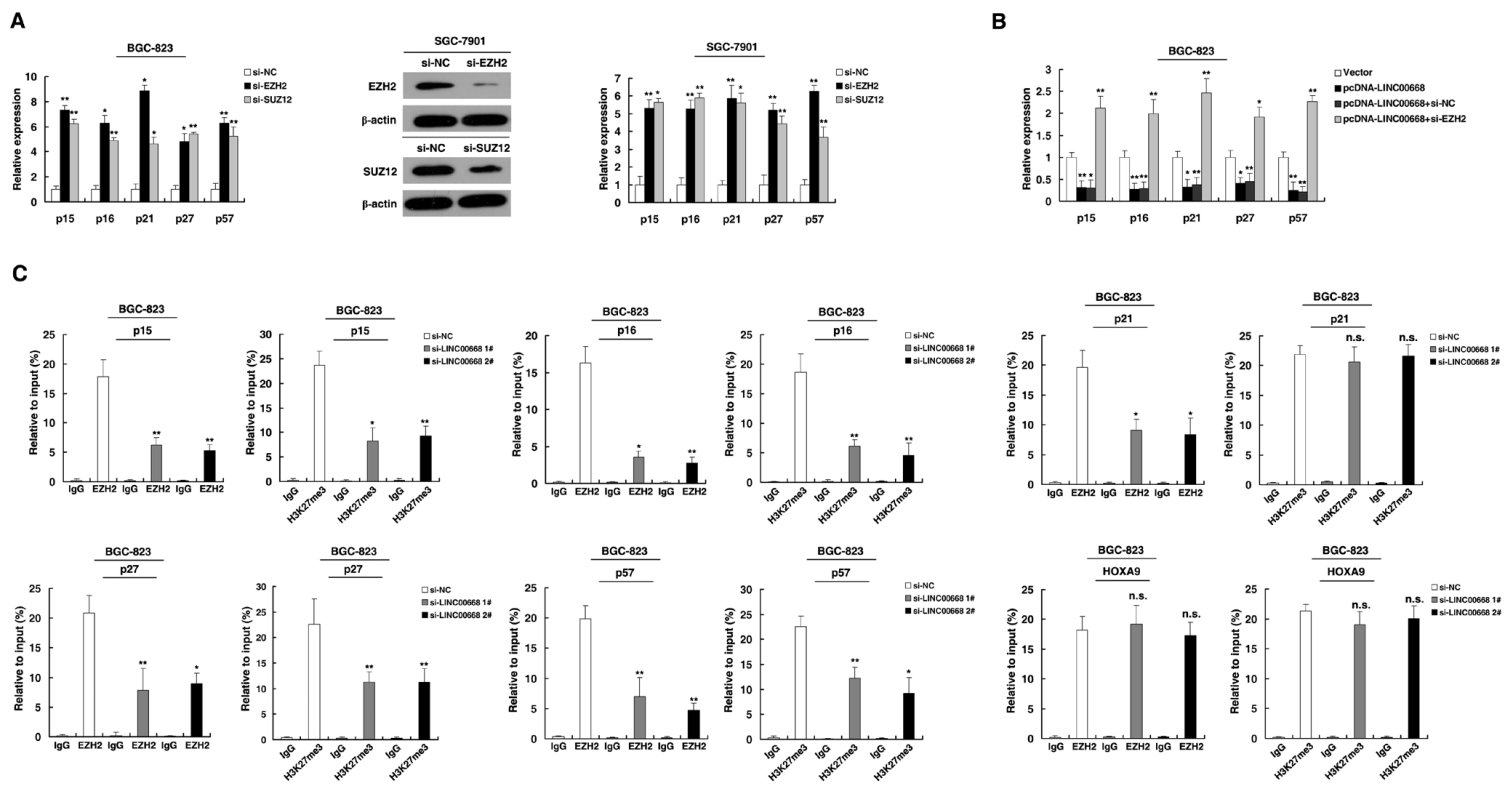
LINC00668 is required to target PRC2 occupancy and activity to epigenetically regulate the expression of CKIs, thus regulating the gastric cancer cell cycle and proliferation **A.** The expression of p15, p16, p21, p27 and p57 in BGC-823 and SGC-7901 cells after knockdown with EZH2 and SUZ12. Western blot assays detected the expression EZH2 and SUZ12 after si-RNA transfection in SGC-7901 cells. **B.** Relative expression was determined in LINC00668-overexpressing BGC-823 cells and cells that were simultaneously transfected with EZH2 siRNA by qRT-PCR. **C.** ChIP-qPCR of H3K27me3 and EZH2 of the promoter region of the p15, p16, p21, p27 and p57 loci after siRNA treatment targeting si-NC or si-LINC00668 in BGC-823 cells. Antibody enrichment was quantified relative to the amount of input DNA. An antibody directed against IgG was used as a negative control. *P < 0.05, **P < 0.01. n.s., not significant.

The role of PRC2 in coregulating suppression of these LINC00668-suppressed CKIs was investigated by EZH2 knockdown, and both were induced in cells transfected with siEZH2 (Figure 5A). Similar results were observed for knockdown of SUZ12 (Figure 5A and Figure S1C). To avoid off-target effects, we used an effective interference target sequence of EZH2 and SUZ12, according to previous study [31, 32]. The PRC2 target genes mentioned above were diminished when LINC00668 was overexpressed. Furthermore, the suppression of genes by LINC00668 was reversed when EZH2 expression was simultaneously down-regulated (Figure 5B). To address whether LINC00668 is involved in transcriptional repression through enrichment of EZH2 to target gene promoters, we conducted ChIP analysis by LINC00668-knockdown. ChIP assays demonstrated that LINC00668 decreased the binding of EZH2 and H3K27me3 levels across the p15, p16, p27 and p57 promoters (Figure 5C). Interestingly, we found that knockdown of LINC00668 decreased the binding of EZH2, but there was no change in H3K27me3 levels across the promoter of p21. These data suggest that EZH2 may silence p21 expression in other ways [33]. As a positive control, no significant change was detected at the promoter of HOXA9, a gene Suppressed by EZH2 [34].

**Figure 6:**
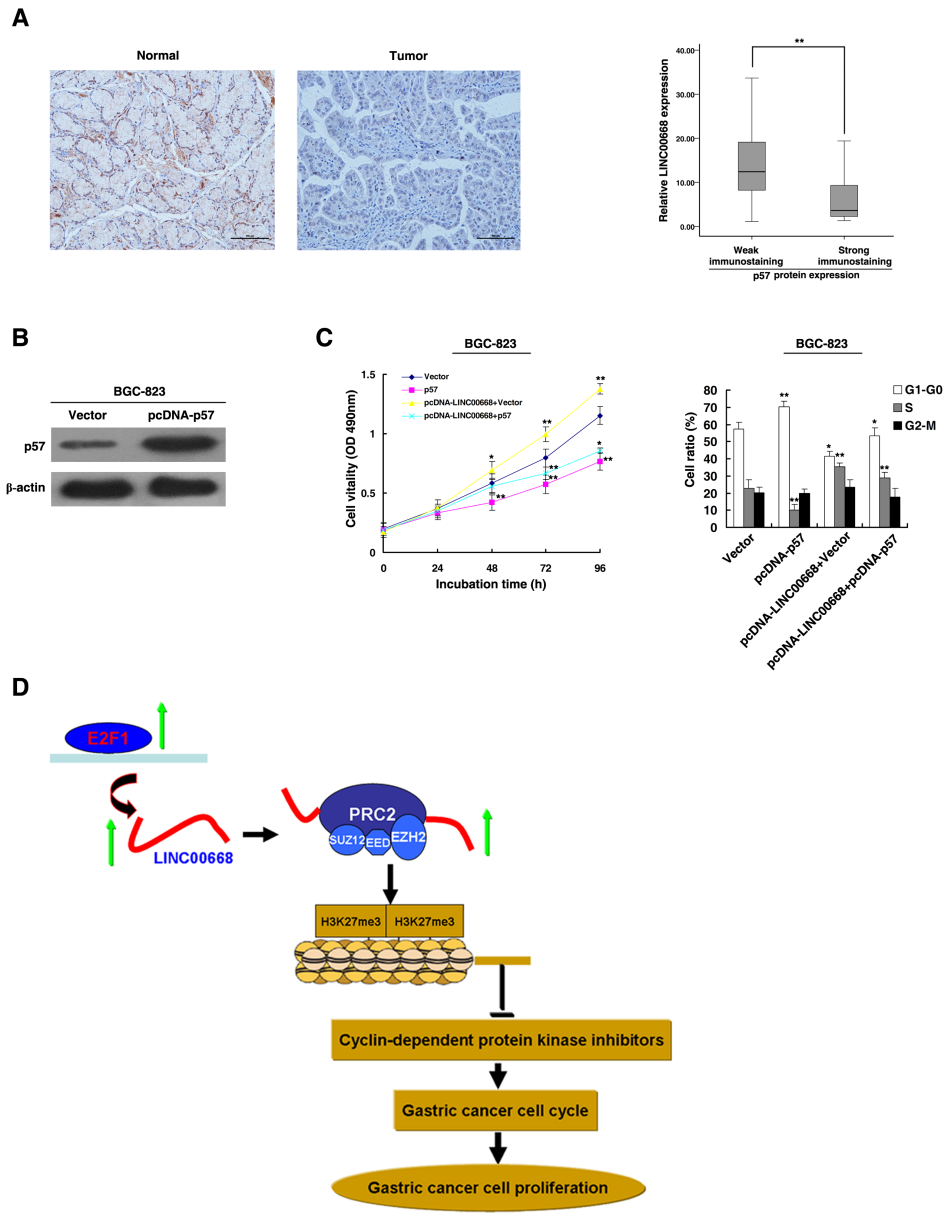
The role of p57 in gastric cancer **A.** Immunohistochemistry was used to detect the expression of p57 protein in 30 GC pairs and the corresponding non-tumor tissues. Bar, 100μm. The immunoreactivity of the p57 protein in GC tissues showed a statistically significant negative correlation with the relative level of LINC00668 expression. **B.** Western blot assays detected the expression of p57 after the transfection of the overexpression plasmid. **C.** BGC-823 transfected with Vector/p57/p57_+_ Vector and transfected with LINC00668 followed by transfection with p57. Forty-eight hours after transfection, the cells were analyzed by MTT assays and flow cytometry. **D.** A proposed model of gastric cancer cell cycle progression and growth control that is mediated by E2F1-regulated LINC00668.*P < 0.05, **P < 0.01.

The functional roles of p15, p16 and p21 have been illustrated in gastric cancer [35, 36]. Our previous research demonstrated that p27 served as a tumor suppressor in gastric cancer [37]. However, the functional role of p57 in GC remains unclear. First, immunohistochemistry was used to detect the expression of p57 protein in 30 paired GC and corresponding non-tumor tissues. The representative results were shown in Figure 6A. P57 was downregulated in GC tissues and was negatively correlated with LINC00668 expression. In addition, overexpression of p57 could induce growth inhibition and G1-G0 phase arrest (Figure 6B and 6C). Overexpression of p57 could partially reverse LINC00668-mediated growth promotion and cell cycle acceleration (Figure 6C).

## DISCUSSION

In our present study, we found that the average level of LINC00668 in GC tissues was significantly higher than those in corresponding non-tumor tissues. The high expression level of LINC00668 in GC was positively correlated with invasion depth and TNM stage. Moreover, high LINC00668 expression in GC tissues was associated with a poor prognosis and could be an independent prognostic indicator. These results suggested that LINC00668 might exhibit important roles in GC progression. In addition, many aberrant expression patterns of lncRNAs have been reported to be involved in the progression of multiple tumors types and can be used as prognostic indicators [12, 38, 39]. Our previous studies also showed that lncRNAs ANRIL and HOTAIR could serve as a prognostic factors in GC [15, 19].

The dysregulation of lncRNAs joins various pathological processes, similar to protein coding transcripts, the transcription of lncRNAs is subject to typical epigenetics-mediated and transcription factor-mediated regulation [11, 13]. Our previous research demonstrated that EZH2 could epigenetically suppress lncRNA SPRY4-IT1 in lung cancer, and p53 could activate lncRNA TUG1 at the transcriptional level [40,41]. In this study, we found that LINC00668 was a direct target of E2F1. These results indicated that overexpression of E2F1 may partly contribute to the upregulation of LINC00668 in GC.

E2F1 plays pivotal roles in cell cycle progression[21]. In addition, overexpression of E2F1 was observed in various types of tumors, including GC [22, 23]. Our results showed that LINC00668 is a cell cycle regulator of GC. Importantly, knockdown of LINC00668 could abrogate E2F1 induced cell cycle acceleration and proliferation promotion. These data indicate that LINC00668 may be a downstream effector of E2F1 and can control gastric cancer cell proliferation by altering the cell cycle.

In human cancer, the pRB-mediated repression of E2F1 is often disrupted through either inactivating the RB1 gene itself, overactivation of cyclinD-CDK4/6 kinases, or inactivation of CDK inhibitors (CKIs). These changes result in inappropriate release of E2F1, and consequently lead to cell cycle disorders and boosting cell proliferation [22]. The kinase activity of Cdk/cyclin complexes is tightly modulated by Cdk inhibitors (CKIs), which serve as brakes to halt cell cycle progression [42]. Cyclin-dependent kinases (Cdks) are serine/threonine kinases and their catalytic activities are modulated by interactions with cyclins and Cdk inhibitors (CKIs). Close cooperation between this trio is necessary for ensuring orderly progression through the cell cycle. Our results demonstrated that knockdown of LINC00668 could induce the expression of CKIs in an EZH2-dependent manner. Many lncRNAs modulate specific gene loci through recruiting and binding to PRC2 protein complexes, and PRC2-mediated epigenetic regulation plays a crucial role in the process of tumor development [12]. CKIs act as tumor suppressors in various cancers, and aberrant methylation in gene promoter regions of CKIs has been linked to downregulation of gene expression [43]. In addition, PRC2-mediated histone methylation contributes to the repression of CKIs [33, 44–47]. Our results explained how CKIs are specifically suppressed by PRC2, due in part through LINC00668.

The functional roles of p15, p16 and p21 have been illustrated in gastric cancer [35, 36]. Our previous research indicated that p27 served as a tumor suppressor in gastric cancer [36]. However, the functional role of p57 in GC remains unclear. Our results found that p57 could serve as a tumor suppressor in GC and that overexpression of p57 could partly reverse LINC00668-mediated growth promotion and cell cycle acceleration.

Many studies have demonstrated that the cell cycle could be regulated by lncRNAs [48]. Our study identified a novel lncRNA-mediated regulator of the gastric cancer cell cycle. LINC00668 may be a downstream effector of E2F1 by binding to PRC2, thus enriching a mechanistic link between lncRNA and the E2F1-mediated cell cycle regulation pathway (Figure 6D). LINC00668 may serve as a target for new therapies in GC.

## MATERIALS AND METHODS

### Expression profiling data retrieval and analysis of lncRNAs in gastric cancer

Gastric cancer lncRNAs expression profiling data was retrieval and analysis. Raw microarray data was downloaded from GEO (GSE53137). Normalized signal data were downloaded and z-score-transformed. Paired *t*-test according to their experimental design was employed to validate probe statistical significance. We use local BLAST+ to map probing sequences to human lncRNA RefSeq database at NCBI. FDR was calculated by Benjamini-Hochberg method.

### Tissue collection and ethics statement

A total of 106 patients analyzed in this study underwent resection of the primary gastric cancer at the First Affiliated Hospital of Nanjing Medical University. The study was approved by the Research Ethics Committee of Nanjing Medical University (Nanjing, Jiangsu, PR China), and written informed consent was obtained from all patients. The clinicopathological characteristics of the gastric cancer patients are summarized in Table 1.

### Cell culture

Three gastric cancer cell lines (SGC-7901, BGC-823, MGC-803), and a normal gastric epithelium cell line (GES-1) were purchased from the Institute of Biochemistry and Cell Biology of the Chinese Academy of Sciences (Shanghai, China). Cells were cultured in RPMI 1640 or DMEM (GIBCO-BRL) medium supplemented with 10 % fetal bovine serum (10 % FBS), 100 U/ml penicillin, and 100 mg/ml streptomycin in humidified air at 37¼C with 5% CO2.

### Cell proliferation assays

Cell proliferation was monitored using the Cell Proliferation Reagent Kit I (MTT) (Roche, Basel, Switzerland). The transfected cells were plated in 96-well plates (3000 cells/well). Cell proliferation was determined every 24 h following the manufacturer's protocol. For the colony formation assay, a certain number of transfected cells were placed into each well of a six-well plate and maintained in media containing 10% FBS for 2 weeks. Medium was replaced every 4 days. Colonies were fixed with methanol and stained with 0.1% crystal violet (Sigma-Aldrich, St. Louis, MO, USA) in PBS for 15 min. The colony formation was determined by counting the number of stained colonies. Triplicate wells were measured in each treatment group.

### RNA extraction and qRT-PCR analyses

Total RNA was extracted from tissues or cultured cells using TRIzol reagent (Invitrogen, Carlsbad, CA). For qRT-PCR, RNA was reverse transcribed to cDNA by using a Reverse Transcription Kit (Takara, Dalian, China). Real-time PCR analyses were performed using SYBR Green (Takara, Dalian China). The results were normalized to the expression of *β-actin*. Primer sequences are listed in Supplementary Table S1.

### Western blot assay and antibodies

Cells protein lysates were separated by 10% SDS-polyacrylamide gel electrophoresis (SDS-PAGE), transferred to 0.22μm NC membranes (Sigma) and incubated with specific antibodies. Autoradiograms were quantified by densitometry (Quantity One software; Bio-Rad). *β-actin* antibody was used as control. Anti-E2F1 and anti-p57 were purchased from Cell Signaling Technology, Inc.

### Flow-cytometric analysis

Cells were harvested after transfection. For cell-cycle analysis, cells were stained with propidium oxide by the Cycle TEST PLUS DNA Reagent Kit (BD Biosciences) following the manufacturer's protocol and then analyzed by flow cytometry (FACScan; BD Biosciences). The percentage of cells in G0-G1, S, and G2-M phase were counted and compared. All experiments were repeated at least three times.

### Xenograft study

Five-week-old athymic BALB/c mice were maintained under specific pathogen-free conditions and manipulated according to protocols approved by the Shanghai Medical Experimental Animal Care Commission. BGC-823 cells were transfected with Scramble or shLINC00668. After 48 h, cells were collected and injected into either side of the posterior flank of the nude mouse. Tumor volumes were examined every 2 days when the implantations were starting to enlarge. Tumor volumes and weights were measured every two days in mice from the control (six mice) or shLINC00668 (six mice) groups, tumor volumes were calculated using the equation length × width^2^ × 0.5. Sixteen days after injection, the mice were sacrificed and tumor weights were measured.

### Transfection of gastric cancer cells

Gastric cancer cells were transfected with siRNA oligonucleotides and plasmids using Lipofectamine 2000 (Invitrogen, USA) according to the manufacturer's protocol. The nucleotide sequences of siRNA for LINC00668 were: siRNA1# sense 5′- CCACUCCCAUCCACUGUAGUGUAAA-3′ and antisense 5′-UUUACACUACAGUGGAUGGGAGUGG-3′; siRNA2# sense 5′-GAAUCUUGGGCGGUCUGAAAUCUGA-3′ and antisense 5′-UCAGAUUUCAGACCGCCCAAGAUUC-3′. Specific siRNA target E2F1, EZH2 and SUZ12 was described previously [49–51]. Typically, cells were seeded at six-well plates and then transfected the next day with specific siRNA (100 nM) and control siRNA (100 nM) by using Lipofectamine 2000. Negative control siRNA (si-NC) were purchased from Invitrogen (Invitrogen, USA). The shLINC00668 was cloned into pENTR™/U6 vector, and the sequence is shown in Supplementary Table S1. The pcDNA-E2F1 expression clone was a kind gift from Zhongmin Yuan (Department of Pharmacology, Zhongshan School of Medicine, Sun Yat-sen University). The sequence of p57 was synthesized and subcloned into a pCDNA3.1 vector (Invitrogen, Shanghai, China). After transfection, cells were harvested for further studies.

### Subcellular fractionation location

Separation of the nuclear and cytosolic fractions was performed using the PARIS Kit (Life Technologies, Carlsbad, CA, USA) according to the manufacturer's instructions.

### Immunohistochemistry

Immunohistochemistry was performed as previously described [52]. Anti-E2F1 and anti-p57 were purchased from Abcam.

### Luciferase reporter assay

The luciferase assays were performed using a luciferase assay kit (Promega) according to the manufacturer's protocol. Briefly, cells were first transfected with appropriate plasmids in 24-well plates. Then, the cells were harvested and lysed for luciferase assays 48 h after transfection. The relative luciferase activity was normalized to renilla luciferase activity. The promoter region of LINC00668 was PCR-amplified by TaKaRa LA Taq (Takara) using LINC00668-p-F (Kpn1 site) and LINC00668-p-R (Xho1 site) primers and was then subcloned into the pGL3 basic firefly luciferase reporter. The amplified PCR fragments were then used as a template for generating promoter constructs carrying deletions using respective primers. All PCR products were verified by DNA sequencing. All primers sequences are summarized in Supplementary Table S1.

### Chromatin immunoprecipitation (ChIP) assays

ChIP assays were performed using the EZ-CHIP KIT according to the manufacturer's instructions (Millipore, USA). EZH2 and SUZ12 antibodies were obtained from Abcam. E2F1 antibody was obtained from Cell Signaling Technology. H3 trimethyl Lys 27 antibody was obtained from Millipore. The ChIP primer sequences are listed in Supplementary Table S1. Quantification of immunoprecipitated DNA was performed using qPCR with SYBR Green Mix (Takara). ChIP data were calculated as a percentage relative to the input DNA by the equation 2^[Input Ct- Target Ct]^ × 0.1×100.

### RNA immunoprecipitation (RIP)

RNA immunoprecipitation (RIP) experiments were performed using a Magna RIP™ RNA-Binding Protein Immunoprecipitation Kit (Millipore, USA) according to the manufacturer's instructions. For RIP assays, antibodies for EZH2, SUZ12 WDR5, LSD1, SETDB1, SUV39H1, DNMT1, DNMT3a and DNMT3b were obtained from Abcam.

### Statistical analysis

All statistical analyses were performed using SPSS 20.0 software (IBM, SPSS, USA). The significance of differences between groups was estimated by Student's *t*-tests and χ2 tests as appropriate. OS rates were calculated by the Kaplan-Meier method with the log-rank test applied for comparison. Survival data were evaluated using univariate and multivariate Cox proportional hazards models. Variables with a value of *p* < 0.05 in univariate analysis were used in subsequent multivariate analysis on the basis of Cox regression analyses. Two-sided p-values were calculated, and a probability level of 0.05 was chosen for statistical significance.

## SUPPLEMENTARY MATERIAL FIGURE AND TABLE




