# Supplementary material for: E2F1-induced upregulation of long noncoding RNA LINC00668 predicts a poor prognosis of gastric cancer and promotes cell proliferation through epigenetically silencing of CKIs
Source: Oncotarget. 2015 Dec 23;7(17):23212–26. doi: 10.18632/oncotarget.6745 (PMC5029621; doi:10.18632/oncotarget.6745)
Supplement: Supplementary file 1 [file oncotarget-07-23212-s001.pdf]

E2F1-induced upregulation of long noncoding RNA LINC00668 predicts a poor prognosis of gastric cancer and promotes cell proliferation through epigenetically silencing of CKIs

Supplementary Material

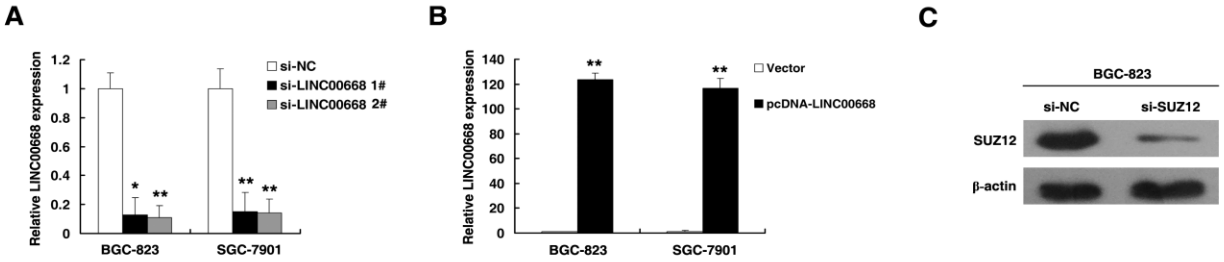

FigureS1 (A) And (B) qRT-PCR was performed to detect the LINC00668 expression after knockdown and overexpression. (C) Western blot assays detected the expression SUZ12 after si-RNA transfection.
